# Lymphangioma circumscriptum in the scrotum: a case report

**DOI:** 10.1186/1752-1947-6-233

**Published:** 2012-08-09

**Authors:** Saroona Haroon, Sheema H Hasan

**Affiliations:** 1 Department of Pathology and Microbiology, Aga Khan University Hospital, Stadium Road, P.O. Box 3500, Karachi, 74800, Pakistan; 2 Department of Pathology and Microbiology, Aga Khan University Hospital, Karachi, 74800, Pakistan

**Keywords:** Follow-up, Lymphangioma circumscriptum, Scrotum, Surgical treatment

## Abstract

**Introduction:**

Lymphangioma circumscriptum is a rare benign skin disorder involving hamartomatous lymphatic malformation of deep dermal and subcutaneous lymphatic channels. It is a therapeutic challenge for the dermatologist when it occurs at common sites such as axilla, shoulder, groin and buttocks and a diagnostic challenge for the surgeon when it occurs at rare sites such as the scrotum. Surgical treatment is the most commonly used method to treat scrotal lymphangioma circumscriptum but there are high rates of recurrence.

**Case presentation:**

We report the case of a 30-year-old Pakistani man who presented with scrotal swelling which was clinically misinterpreted as an infectious disorder. Later on re-resection of deeper tissue was performed to prevent recurrence. He is still being followed-up on a regular basis.

**Conclusion:**

Awareness of the occurrence of lymphangioma circumscriptum in the scrotum in adult men without prior disease is mandatory to avoid missing the diagnosis and to ensure proper treatment.

## Introduction

Lymphangioma circumscriptum (LC) is a rare benign skin disorder involving hamartomatous lymphatic malformation of deep dermal and subcutaneous lymphatic channels. Fluid-filled vesicles that contain lymphatic fluid are typically seen in patients with LC. Scrotal LC is very rare and may present as a congenital condition or, rarely, might develop secondary to radiotherapy, infection, or surgery such as a vasectomy. Several medical and abrasive treatment modalities are available including laser therapy. Surgical resection is the most commonly used method to treat scrotal LC but there are high rates of recurrence. Here we report the case of a patient who presented with scrotal swelling which was clinically misinterpreted as an infectious disorder. This clinical misdiagnosis points to the fact that histopathology is necessary even if clinically the surgeon suspects an inflammatory disorder. The patient had no significant past medical or surgical history and the cause of his rare scrotal LC remained unidentified. Re-resection of deeper tissue was performed later because LC is a disease with high recurrence rates. He is still being followed-up on a regular basis.

## Case presentation

**Figure 1:**
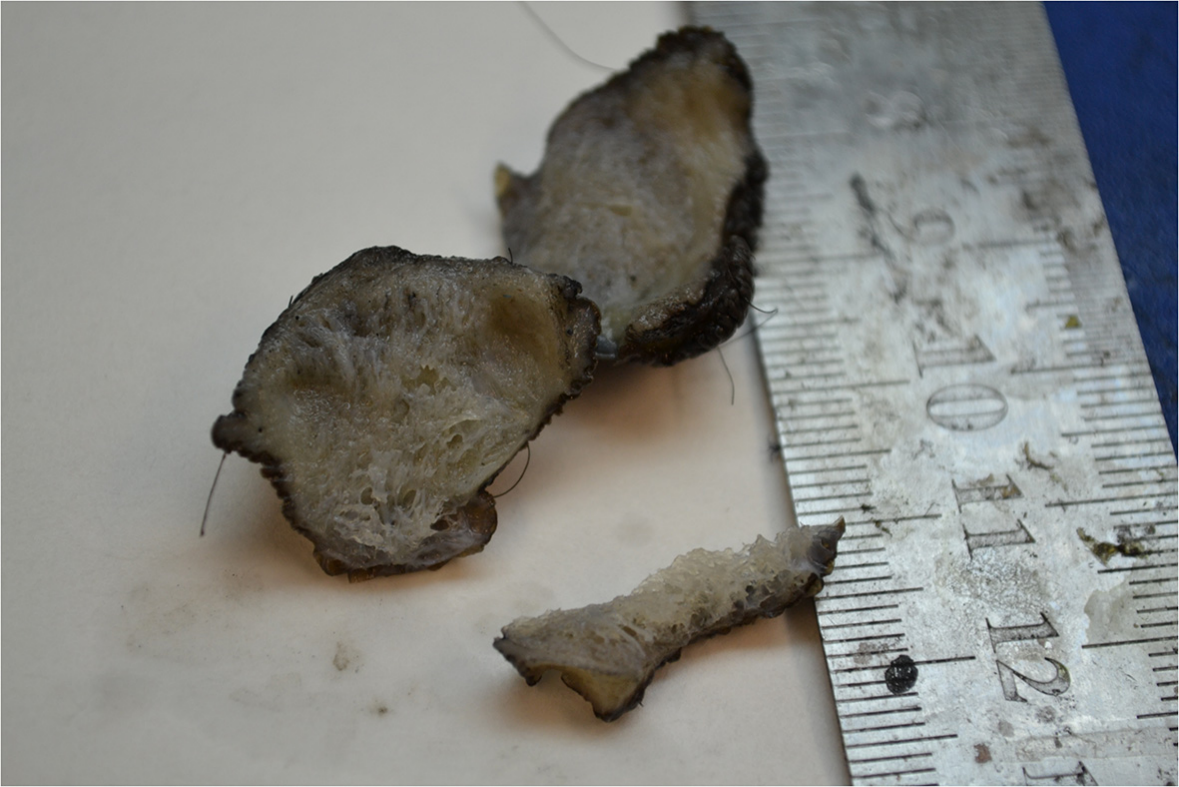
Gross picture of scrotal lymphangioma circumscriptum, skin covered tissue with tiny cystic spaces.

**Figure 2:**
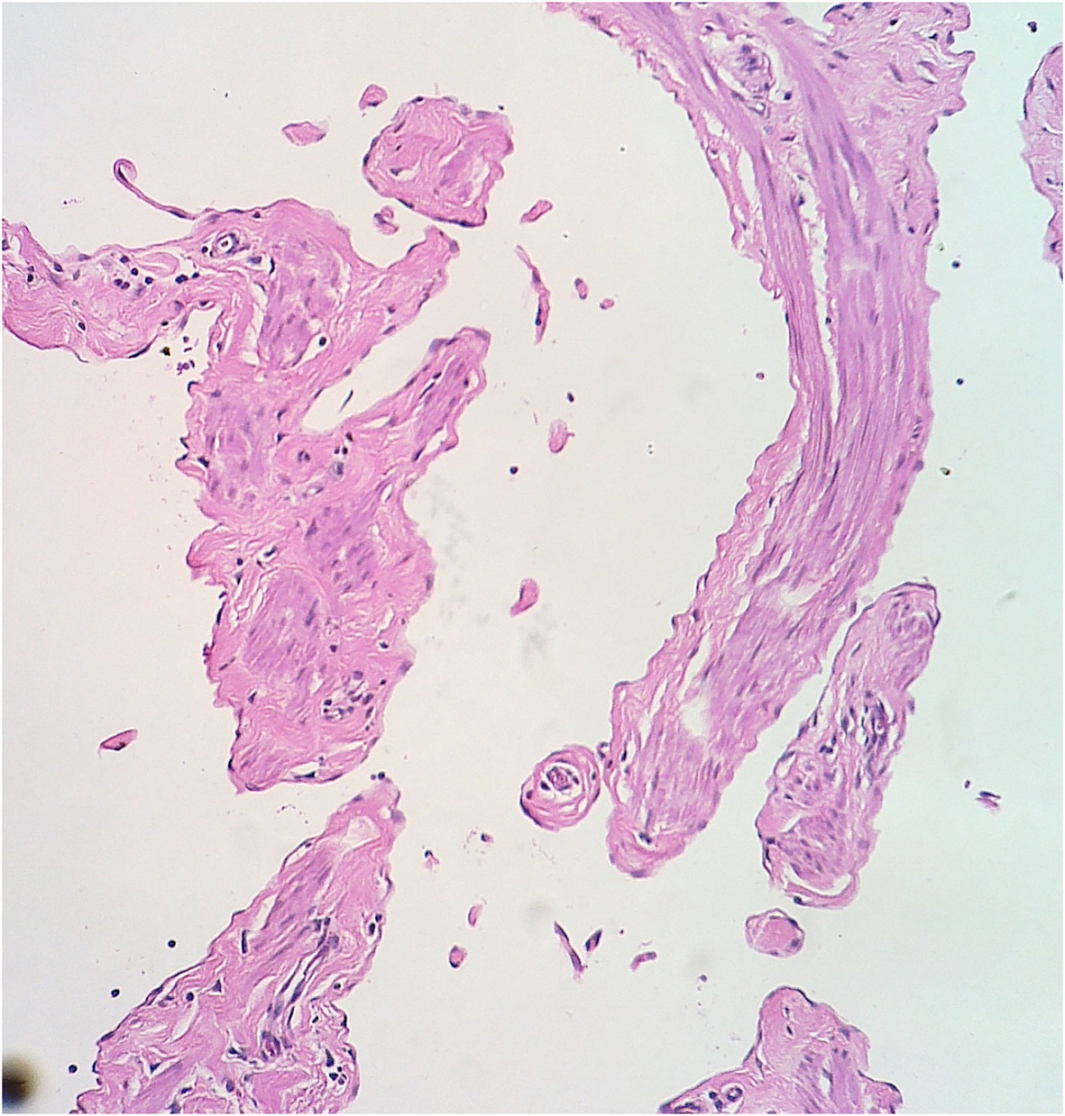
Microscopic picture of lymphangioma circumscriptum with widely spaced vascular channels (hematoxylin and eosin, 20×).

**Figure 3:**
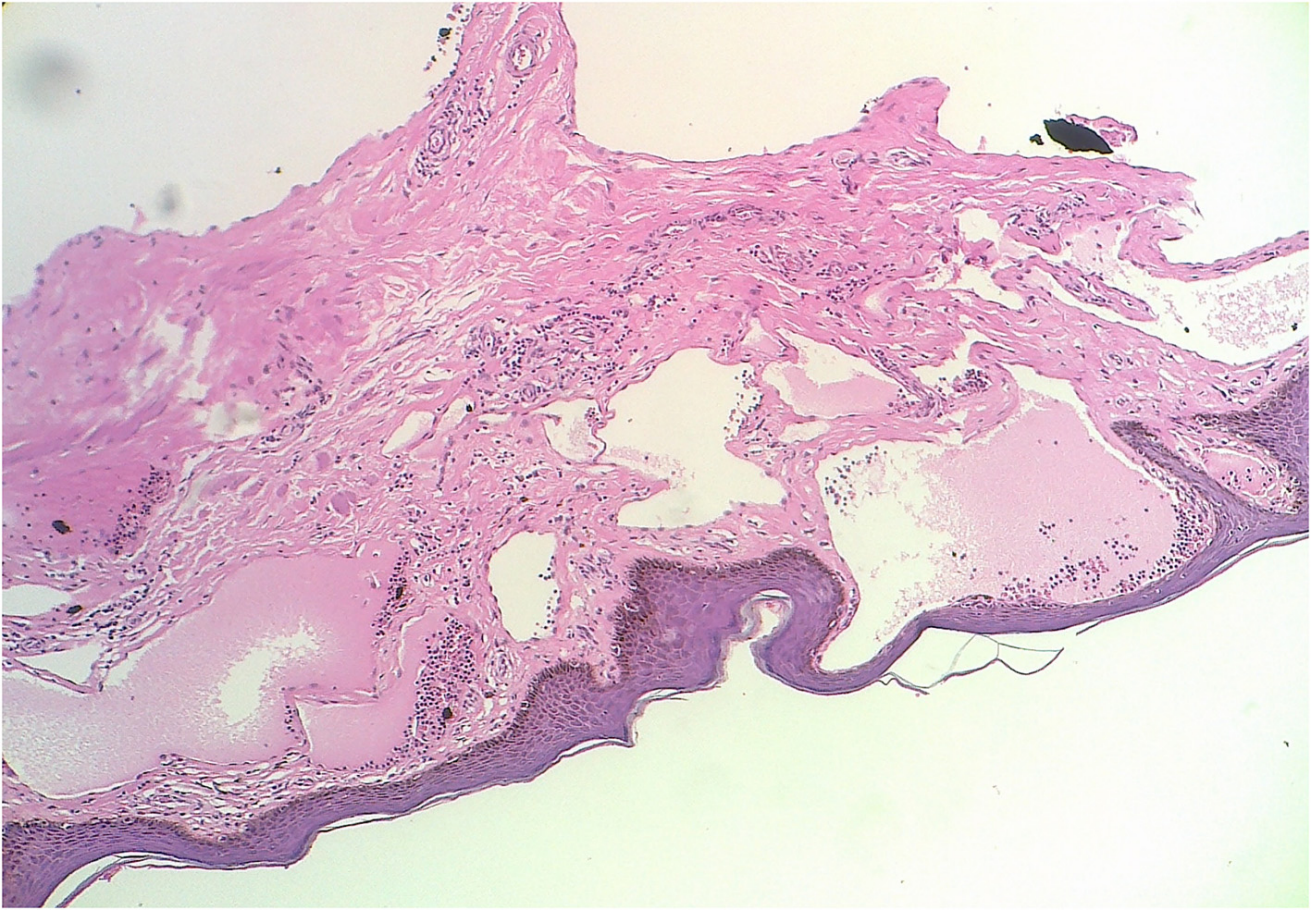
Microscopic picture with spaces having lining of flattened endothelial cells present just beneath the skin (hematoxylin and eosin, 20×).

A 30-year-old Pakistani man presented to our general surgery outpatient department with a complaint of a slow growing painless scrotal swelling present for the past two years. His lesion was itchy and no discharge was present. On examination there was a soft copper-colored swelling involving the medial, lower and lateral sides of his scrotum. Clinical diagnosis of an infectious disorder such as molluscum contagiosum was made. He had no significant past medical history of sexually transmitted disease or surgical procedure in that particular area. He had a normal laboratory checkup including blood biochemistry, serum lactate dehydrogenase levels and serology for sexually transmitted diseases and filariasis. There was no peripheral eosinophilia. Detailed physical examination, serology and the absence of eosinophilia excluded the possibility of the common etiology, filariasis. Surgical resection was performed and the specimen was sent for histopathology. The tissue was skin covered, measured 2.5cm × 2.5cm and the skin had a soft nodular raised area measuring 1.5cm × 1.5cm (Figure 1). The histopathological examination revealed a very rare disorder of the scrotum with dilated thin walled lymphatic channels just beneath the skin (Figures 2 and 3). Re-resection of deeper tissue was advised, which was undertaken and he had post-operative antibiotics. He has been free of complaints during five post-operative months.

## Discussion

LC or microcystic lymphatic malformation are the terms used for hamartomatous abnormality of the lymphatic channels of the skin, which can be encountered anywhere in the body. The predominant types of lymphangiomas are LC, cavernous lymphangioma, cystic hygroma and lymphangioendothelioma. These conditions account for approximately 26% of benign vascular tumors in children but are rarer in adults. The most common form of cutaneous lymphangioma is LC which arises in infancy but may occur at any age [1,2]. Clinical findings consist of many vesicles that are pink to copper-colored and can be red or black if there is secondary hemorrhage in the vesicles. These vesicles contain lymph and are often compared with frog spawn. LC is commonly found on the axillary folds, shoulders, neck, proximal limbs, and oral cavity with the scrotum being the rarest site. LC is asymptomatic; however, it can be complicated by excessive drainage and recurrent cellulitis [3].

Whimster first proposed the pathogenesis of LC in 1976. Whimster postulated a collection of subcutaneous lymph cisterns, which arise during embryonic development, that are not connected to the lymphatic system and therefore unable to drain the lymph received from surrounding tissue. The cisterns are lined with muscle that contracts and, by applying pressure, produces protrusions on the skin [4]. Acquired LC develops in advanced age, possibly due to injury to deep collecting lymphatics, caused by radiotherapy damage or infections such as filariasis, lymphogranuloma venereum, or tuberculosis. In our patient, however, there was no history of radiotherapy or infections [3,5].

The definitive diagnosis is usually made by biopsy because these lesions often mimic infectious diseases such as molluscum contagiosum and filariasis; there was clinical misinterpretation of the symptoms in our case. Histopathology of LC revealed dilated lymph vessels with a lining of flat endothelial cells, mostly in the upper dermis and subcutis (Figures 2 and 3). There was no endothelial swelling depicting lymphangitis, granulomatous reaction or adult filarial worms, which can be present in filariasis.

The disorder is clinically identified by translucent or hazy vesicles of different sizes which are grouped like frog spawn or, less commonly, as diffuse swelling to a particular area. These swellings frequently have accompanying verrucous alterations giving them a warty appearance and, if there is significant hyperkeratosis, the swelling may clinically resemble condyloma acuminata. Sometimes the epidermis shows acanthosis and hyperkeratosis with widening of papillary dermis. The deeper dermis shows wide ectatic channels with a lining of endothelium containing lymph [6,7].

Indications for treatment of the clinically evident LC include both its cosmetic appearance and prevention of complications such as cellulitis. The definitive treatment for lymphangiomas is surgical excision of both the superficial component, the skin, and the deeper component, the subcutaneous tissue which has feeding cisterns. In our case there is a deeper component reaching the resection margin which could lead to recurrence. The most common postoperative complication is recurrence of LC with an incidence of 25% to 50% within three months, which is usually due to an improper surgical approach or inadequate excision of the tumor [6,8]. The scrotal reconstruction was done by free skin graft. Other palliative management modalities incorporate a wide variety of options such as surface abrasion by X-ray therapy, radiotherapy, argon laser, CO^2^ laser, 900-nm diode laser, pulsed dye-laser, and sclerotherapy and observation.

## Conclusions

Awareness of the occurrence of LC in the scrotum in adult men without prior disease is important to avoid missing the diagnosis, and to prevent inappropriate treatments, because its clinical presentation is similar to infectious disorders. Currently, surgery is an effective, well-tolerated, efficient, and easily applicable treatment. Incomplete excision is the most common reason for recurrence after surgical treatment [3,6]. Complete excision of involved areas is important to prevent recurrence. Involvement of tissues as shown by radiology and histopathology is generally wider and deeper compared to clinical assessment. Therefore, excision should include all subcutaneous tissues down to the fascia.

## Consent

Written informed consent was obtained from the patient for publication of this manuscript and any accompanying images. A copy of the written consent is available for review by the Editor-in-Chief of this journal.

## Competing interests

The authors declare that they have no competing interests.

## Authors’ contribution

SH collected the patient’s data regarding his past history and follow-up. SH and SHH performed the histological examination of the scrotal tissue and were major contributors in writing the manuscript. All authors read and approved the final manuscript.

## References

[B1] Vlastos AT Malpica A Follen M Lymphangioma circumscriptum of the vulva: a review of the literature Obstet Gynecol 2003 101 946 954 10.1016/S0029-7844(03)00048-6 12738156

[B2] Lapidoth M Treatment of lymphangioma circumscriptum with combined radiofrequency current and 900 nm diode laser Derm Surg 2006 32 790 794 10.1111/j.1524-4725.2006.32162.x 16792643

[B3] Bikowski JB Dumont AMG Lymphangioma circumscriptum: treatment with hypertonic saline sclerotherapy J Am Acad Dermatol 2005 53 442 444 10.1016/j.jaad.2005.04.086 16112350

[B4] Pal DK Banerjee M Moulik D Biswas BK Choudhury MK Lymphangioma circumscriptum of the scrotum following vasectomy Indian J Urol 2010 26 294 295 10.4103/0970-1591.65410 20877614 PMC2938560

[B5] Sachdeva S Lymphangioma circumscriptum treated with radiofrequency ablation Indian J Dermatol 2011 56 77 78 10.4103/0019-5154.77558 21572798 PMC3088942

[B6] Lapidoth M Ackerman L Amital DB Raveh E Kalish E David M Treatment of lymphangioma circumscriptum with combined radiofrequency current and 900 nm diode laser Dermatol Surg 2006 32 790 794 10.1111/j.1524-4725.2006.32162.x 16792643

[B7] Omprakash HM Rajendran SC Lymphangioma circumscriptum (microcystic lymphatic malformation): palliative coagulation using radiofrequency current J Cutan Aesthet Surg 2008 1 85 88 10.4103/0974-2077.44165 20300350 PMC2840899

[B8] Sheu JY Chung HJ Chen KK Lin AT Chang YH Wu HH Huang WJ Hsu YS Kuo JY Chang LS Lymphangioma of male exogenital organs J Chin Med Assoc 2004 64 204 206 15244022

